# Supplementary material for: Rapid and efficient hydrogen clathrate hydrate formation in confined nanospace
Source: Nat Commun. 2022 Oct 10;13:5953. doi: 10.1038/s41467-022-33674-2 (PMC9550858; doi:10.1038/s41467-022-33674-2)
Supplement: Supplementary file 1 — Supplementary Information [file 41467_2022_33674_MOESM1_ESM.docx]

Supplementary Information for

**Rapid and efficient hydrogen clathrate hydrate formation in confined nanospace**

Judit Farrando-Perez^1^†, Rafael Balderas-Xicohtencatl^2^†, Yongqiang Cheng^2^, Luke Daemen^2^, Carlos Cuadrado-Collados^1^, Manuel Martinez-Escandell^2^, Anibal J. Ramirez-Cuesta^2^*, Joaquin Silvestre-Albero^1^*

Correspondence to: joaquin.silvestre@ua.es; ramirezcueaj@ornl.gov

**This PDF file includes:**

Supplementary Figs. 1 to 6

Supplementary Tables 1 to 2

**Supplementary Figures**

**Supplementary Fig. 1.** XPS analysis of the synthesized PPAC carbon material in the N*1s*, O*1s*, and C*1s* regions. Source data are provided as a Source Data file.


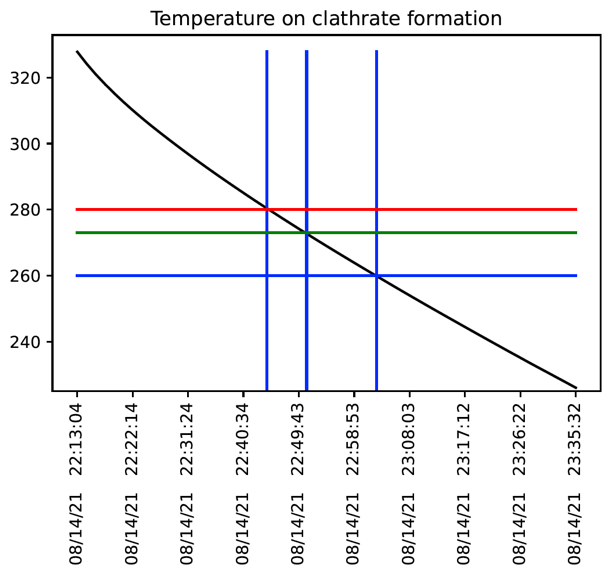

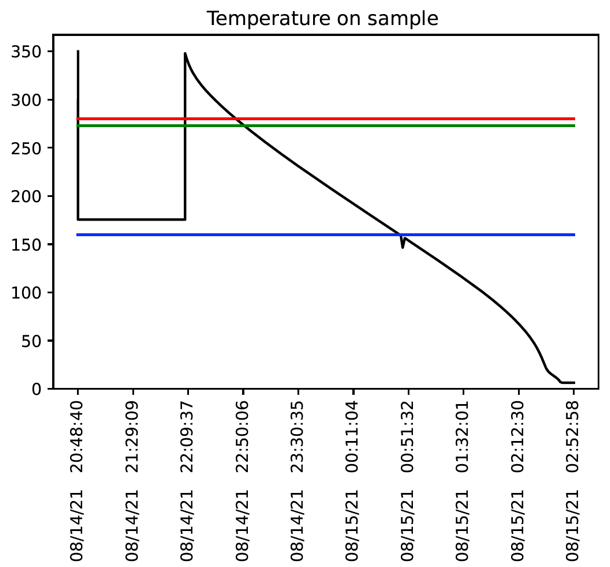


Temperature (K) on sample

Temperature (K) on clathrate formation

**Supplementary Fig. 2.** Temperature profiles were followed during the INS experiments as a function of time. Both figures correspond to the temperature profile used in the D_2_O-PPAC sample under 135 MPa hydrogen. (Left panel) Initially, the sample is heated to 350K at atmospheric pressure. Once the temperature is reached, the cell is loaded with hydrogen (135 MPa) and the system is submitted to a cooling ramp down to 5K. Red and green horizontal lines define the hydrogen clathrate formation temperature region (280K - 273K). Once the temperature reaches 160K (blue line), the sample cell is evacuated down to 0.1 MPa (an endothermic peak can be appreciated). (Right panel) The amplification of this temperature profile clearly shows that the cooling between 280K and 273K is extremely fast (*ca.* 5 minutes), i.e., complete water-to-hydrate formation takes place in less than 10 minutes.

**Supplementary Fig. 3.** INS spectrum of hydrogen clathrate hydrate confined in D_2_O-PPAC formed under 200 MPa. Assignment of the different contributions.

**Supplementary Fig. 4.** INS spectra of the D_2_O-PPAC sample after pressurizing with hydrogen at 135 MPa at 280K and 273K for 90 minutes. After this period, the sample is cooled down to 5K following the temperature and pressure profile described in supplementary Fig. 2. The obtained pattern clearly shows that hydrogen clathrate is formed only at 280K. In other words, liquid D_2_O is needed to promote the hydrate formation process. Source data are provided as a Source Data file.

**Supplementary Fig. 5.** INS spectra of the hydrogen clathrate hydrate confined in D_2_O-PPAC sample and formed under 135 MPa hydrogen pressure and evacuated down to 0.1 MPa (see fig. S2) as a function of sample cell temperature. Note that the rotational features are still observable at 240K (despite the large increase in the Debye-Waller factor). These results confirm that the hydrogen molecules are still enclathrated inside the cages at 240K, in agreement with diffraction data. Source data are provided as a Source Data file.

**Supplementary Fig. 6.** Illustrative scheme of the hydrogen clathrate formation in bulk and confined environments.

**Supplementary Tables**

**Supplementary Table 1.** Chemical composition (at.%) of the PPAC carbon determined from the XPS analysis.

| PPAC | Carbon | Oxygen | Nitrogen | Sulphur |
| --- | --- | --- | --- | --- |
|  | 95.1 | 4.1 | 0.7 | <0.1 |

**Supplementary Table 2.** Comparison of the storage capacity for bulk and confined hydrate crystals.

|  | Bulk H_2_ hydrate | Confined H_2_ hydrate |
| --- | --- | --- |
| Crystal structure | sII | sII |
| Stoichiometry^*^ | 64 H_2_·136 H_2_O | 64 H_2_·136 H_2_O |
| Gravimetric capacity (wt.%) | 5.0 | 4.1 |
| Volumetric capacity (g/L)^**^ | 46.7 | 41.9 |

* Assuming double occupation of small cages and 4 H_2_ molecules in large cages

**Assuming a density for the H_2_ hydrate of 0.94 g/cc
